# Unraveling the Guest‐Induced Switchability in the Metal‐Organic Framework DUT‐13(Zn)**

**DOI:** 10.1002/chem.202100599

**Published:** 2021-05-21

**Authors:** Bodo Felsner, Volodymyr Bon, Jack D. Evans, Friedrich Schwotzer, Ronny Grünker, Irena Senkovska, Stefan Kaskel

**Affiliations:** ^1^ Faculty of Chemistry and Food Chemistry Technische Universität Dresden Bergstraße 66 01069 Dresden Germany

**Keywords:** breathing metal-organic frameworks, DUT-13, flexibility, in situ powder X-ray diffraction

## Abstract

The switching mechanism of the flexible framework Zn_4_O(benztb)_1.5_ (benztb=*N*,*N*,*N’*,*N’*‐benzidine tetrabenzoate), also known as DUT‐13, was studied by advanced powder X‐ray diffraction (PXRD) and gas physisorption techniques. In situ synchrotron PXRD experiments upon physisorption of nitrogen (77 K) and *n*‐butane (273 K) shed light on the hitherto unnoticed guest‐induced breathing in the MOF. The mechanism of contraction is based on the conformationally labile benztb ligand and accompanied by a reduction in specific pore volume from 2.03 cm^3^ g^−1^ in the open‐pore phase to 0.91 cm^3^ g^−1^ in the contracted‐pore phase. The high temperature limit for adsorption‐induced contraction of 170 K, determined by systematic temperature variation of methane adsorption isotherms, indicates that the DUT‐13 framework is softer than other mesoporous MOFs like DUT‐49 and does not support the formation of overloaded metastable states required for negative gas‐adsorption transitions.

## Graphical abstract

**Breathe**! Combining synchrotron in situ PXRD with gas adsorption has shed light on the mechanism of guest‐induced breathing in dynamic DUT‐13 framework. In the contracted‐pore phase, the crystal has pores half the volume of those in the open‐pore phase; this is consistent with the adsorption isotherms. Methane physisorption isotherms at different temperatures indicate the softness of the framework.

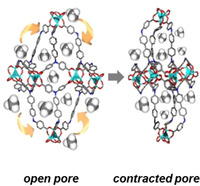

## Introduction

One of the unique features of metal‐organic frameworks (MOFs) is the ability to switch from a porous open state to a less porous, contracted state (breathing), which can be triggered by external stimuli such as guest molecules, temperature, hydrostatic pressure or electromagnetic irradiation.^[1]^ Switchable MOFs are discussed as advantageous for the number of applications, such as adsorptive gas storage,^[2]^ gas separation and sensory applications.^[3]^ Unique mechanical properties suggest using them as shock absorbers or nano dampers.^[4]^

In terms of thermodynamics, switchable MOFs have at least two minima in their free energy profile, considering the Helmholtz free energy of the empty host vs. unit cell volume which are characteristic for a highly porous and a less porous phase respectively. Depending on the positions of these minima, different kinds of switching are expected. In case of so‐called gate‐opening MOFs, the closed pore (*cp*) phase is usually thermodynamically stable in the desolvated state, while the open pore (*op*) phase becomes lower in energy after the pores are filled with guests.^[5]^ After reaching the threshold chemical potential, also denoted as “gate‐opening” pressure, which reflects the activation barrier for the phase transition from *cp* to *op* phase, the MOF starts to adsorb the guests.^[6]^ Desorption of the guests from the pores proceeds at much lower pressures and is driven by thermodynamics. In isothermal gas physisorption, the activation barrier is reflected in the hysteresis between adsorption and desorption branches of the isotherm and can be tuned by variation of the stiffness of the metal nodes, intermolecular interactions, etc.^[7]^ A different situation occurs for breathing mesoporous MOFs, such as the DUT‐49 series (DUT – Dresden University of Technology), which are characterized by a thermodynamically stable *op* phase and contract upon adsorption of guests, because the free energy of the *cp* phase becomes lower at defined guest loading.^[8]^ In addition to breathing, some of these MOFs show negative gas adsorption (NGA), characterized by desorption of the fluid from the pores of the overloaded metastable *op* phase upon adsorption‐induced framework contraction.^[9]^

The mechanism of flexibility in DUT‐49 is based on the buckling of the tetratopic carbazole based ligand, which is characterized by simultaneous change of the interplanar angle between carbazoles and bending of the biphenyl moiety. Hence, increasing the rotational degree of freedom in the ligand molecule may decrease the mechanical stiffness of the framework and broaden the temperature range for breathing and, in particular, NGA for a defined fluid.[^8^, ^10^]

**Figure 1:**
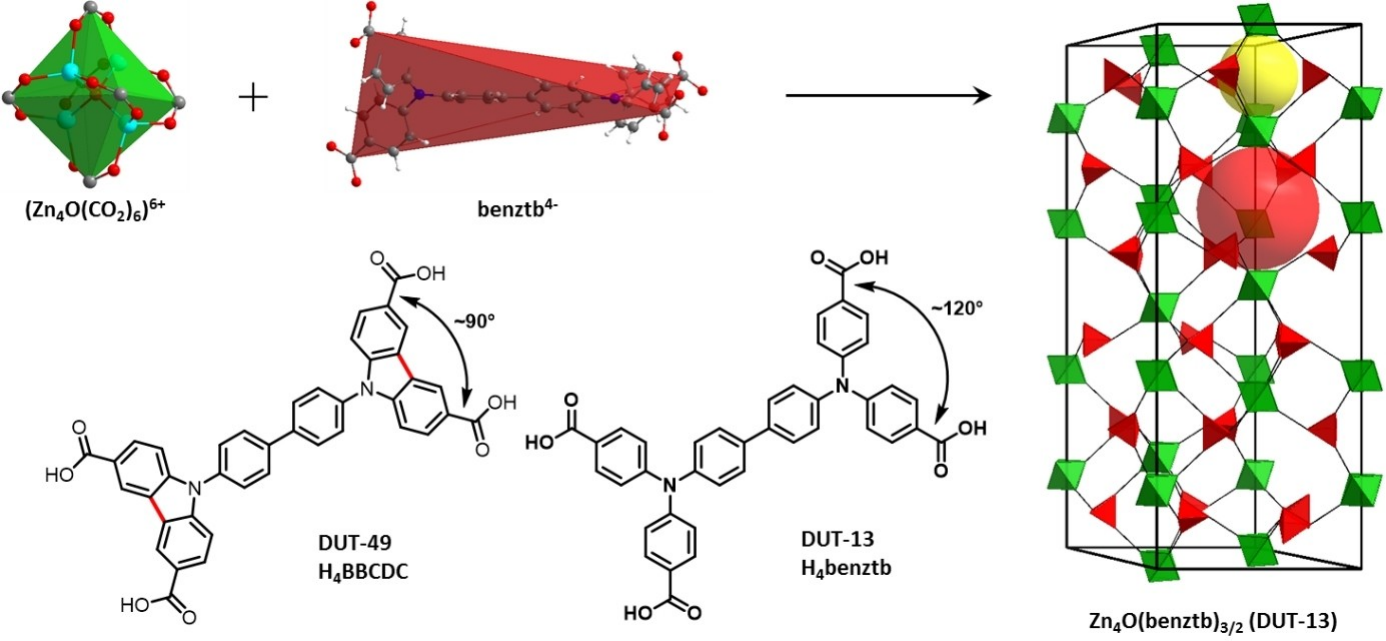
Structure of DUT‐13 simplified as **cor** net (right) with its tetrahedral pore (yellow sphere) and octahedral pore (red sphere) and how it is constructed from the secondary building units Zn_4_O(CO_2_)_6_ (green octahedra) and benztb (red tetrahedra). If the simplified linker tetrahedron is split into two triangles around the Nitrogen's, the structure can also be described as **ttu‐a** topology.^[12]^ Also shown: H_4_BBCDC (DUT‐49 linker, bottom left) and H_4_benztb (DUT‐13 linker, bottom centre).

With this in mind we considered *N*,*N*,*N’*,*N’*‐benzidine tetrabenzoic acid (H_4_benztb) as a ligand, which was used in our group earlier for the design of highly porous MOFs.^[11]^ The molecular structure of the H_4_benztb ligand is remarkably similar to 9,9′‐([1,1′‐biphenyl]‐4,4′‐diyl)bis(9**H**‐carbazole‐3,6‐dicarboxylic acid (H_4_BBCDC) used for the synthesis of DUT‐49. The main difference is in the phenyl rings close to the carboxylate group, namely in case of H_4_BBCDC these two phenyls are interconnected and form 3,6‐carbazole dicarboxylate moiety, whereas in the H_4_benztb linker these two phenyls are connected only to the nitrogen atom (Figure 1). This results in significant differences in the electronic structure and stereochemistry of the ligand. The interconnection of two phenyl rings in H_4_BBCDC leads to the planar 3,6‐carbazoledicarboxylate moiety with nearly 90° angle between the planes of carboxylates, which makes this ligand suitable for the formation of carbazole‐based metal‐organic polyhedrons (MOPs). In case of H_4_benztb, the planar arrangement of substituents around the nitrogen atom is undisturbed and therefore a regular triangle with around 120° bond angles results, which also dominates the angles between the carboxylic groups (Figure 1).

Indeed, a combination of H_4_benztb with paddle wheel dimer leads to the structures of DUT‐10 (Zn_2_(eclipsed‐benztb)(H_2_O)_2_ – space group *Imma*), DUT‐11 (Zn_2_(staggered‐benztb)(H_2_O)_2_ – space group *Cccm*) and DUT‐12 (Cu_2_(benztb)(H_2_O)_2_ – space group *P*4*/mnc*), which could not be desolvated without a phase transition to *cp* phase (DUT‐10) or loss of three‐dimensional order (DUT‐11 and DUT‐12).^[11b]^ Obviously in these cases, mechanical stiffnesses of the frameworks are too low due to the synergetic effects of the highly flexible ligand and the soft hinges to the nodes.^[13]^ The use of the more rigid Zn_4_O^6+^ node in the synthesis resulted in the frameworks Zn_4_O(benztb)_3/2_, known as DUT‐13^[11a]^ or, if additional H_3_BTB was used in the synthesis, Zn_4_O(benztb)(btb)_2/3_ (DUT‐25).^[11c]^ Both frameworks preserve their structures upon desolvation using supercritical CO_2_. While DUT‐25 shows reversible isotherms in nitrogen (77 K) and carbon dioxide (195 K) physisorption experiments, typical for mesoporous MOFs, nitrogen (77 K) and *n*‐butane (273 K) physisorption experiments on DUT‐13 exhibit hysteretic behavior, typical for breathing MOFs. The structure of the *op* phase was reported earlier, however, the flexibility mechanism is not clarified yet. The recent progress in understanding of flexibility in MOFs,^[7d]^ promoted by development of advanced in situ techniques,^[14]^ findings about crystallite size effects,^[15]^ thermodynamic driving forces,^[8]^ study of mechanical properties,^[16]^ adsorption enthalpy and temperature,^[10]^ motivated us for further investigation of flexibility mechanism in DUT‐13.

With this in mind, we analysed the guest‐induced breathing in DUT‐13 upon physisorption of nitrogen (77 K) and *n*‐butane (273 K) by in situ synchrotron PXRD upon adsorption and desorption of the gases. The crystal structure of the contracted phase of DUT‐13, solved from PXRD patterns illustrates the breathing mechanism as confirmed by theoretical calculations. Physisorption of methane, conducted in a broad temperature range, helped to experimentally access the energetic of adsorption induced *op*–*cp* phase transition.

## Results and Discussion

The synthesis of DUT‐13 was successfully reproduced using the earlier reported procedure.^[17]^ The phase purity of the as synthesized and supercritically desolvated powder was confirmed by PXRD and porosity and flexibility of the sample was proven in a nitrogen physisorption experiment at 77 K (Figures S1–S6 in the Supporting Information). SEM images show a broad crystal size distribution, with the crystal sizes ranging from 2.4 to 36.8 μm (Figure S7). The measured size corresponds to the edge length of the rhombohedral crystals.

### In situ PXRD during adsorption of n ‐butane (273 K) and nitrogen (77 K)

**Figure 2:**
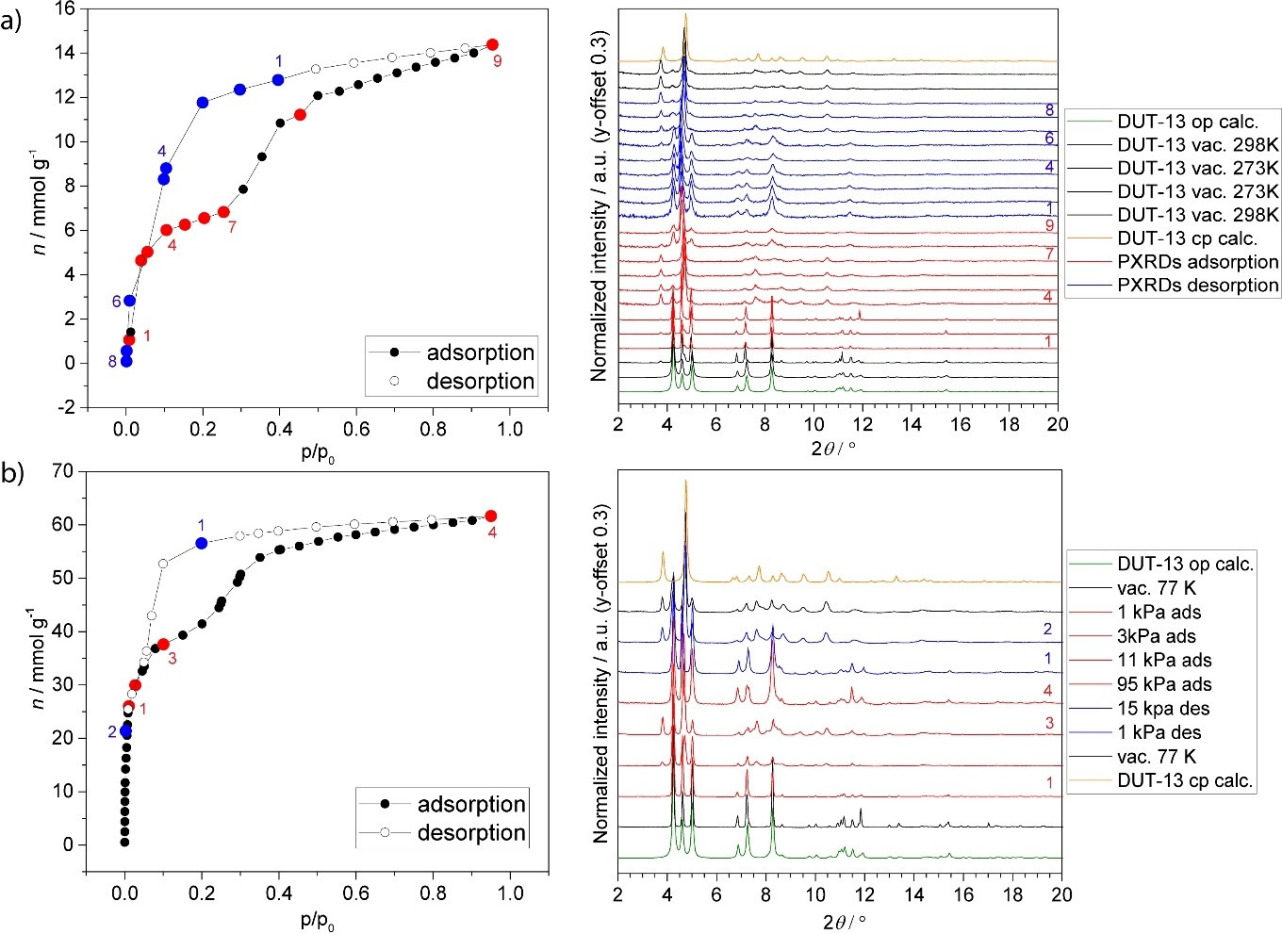
In situ PXRD during the adsorption and desorption of a) *n*‐butane at 273 K and b) nitrogen at 77 K.

Guest‐induced phase transitions in DUT‐13 were monitored by synchrotron PXRD during physisorption of *n*‐butane (273 K) and nitrogen (77 K) in order to understand the switching mechanism and determine the phase composition in the selected points of the isotherm. In both cases in situ measured isotherms exactly reproduced the isotherms, obtained without parallelization (Figure 2). PXRD patterns, measured on the evacuated DUT‐13 framework match well with the theoretical PXRD of the as made structure (Figure S1). The 200 peak at *2θ*=3.8°, characteristic for the *cp* phase, appears in the PXRD patterns in the relative pressure range of 0.03–0.10 (N_2_, 77 K) and 0.10–0.25 (*n‐*C_4_H_10_, 273 K) upon adsorption. Interestingly, in case of nitrogen physisorption at 77 K, a narrower hysteresis loop is observed between adsorption and desorption curves in comparison with the physisorption isotherm of *n*‐butane at 273 K. PXRD patterns, measured at *p/p_0_* =0.11 in the adsorption of the nitrogen (Figure 2b, point 3) indicate a mixture of *op* and *cp* phases. In contrast, PXRD patterns, measured in the plateau of the *n*‐butane isotherm (Figure 2a, points 4 to 7), show mainly reflections of the *cp* phase. The pore volumes, derived at *p/p_0_* =0.11 from the nitrogen and *n*‐butane isotherms, amount to 1.04 and 0.58 cm^3^ g^−1^, respectively, and can be considered as additional indirect proof of the incomplete *op*→*cp* transformation in the case of the nitrogen physisorption. The reason for this could be a broad crystallite size distribution, observed in the DUT‐13 sample (Figure S7), as it has been previously shown for other frameworks that the smaller crystals do not contract.^[15b]^ At relative pressures close to saturation the pure *op* phase is observed indicating a reopening of the structure for both gases. Interestingly, in the case of reopening with *n*‐butane, the reflection intensities in the measured PXRD strongly differ from the theoretical patterns, calculated for the guest‐free MOF, which can be considered as an indication of the ordering of the *n*‐butane molecules in the pores (Figure 2a). Because of the combination of low crystallinity and peak broadening in the *cp* phase, no further structural details on the gas molecules ordering in the pores could be obtained from the Rietveld refinement. Desorption branches in both cases show the reverse *op*→*cp* transition after desorption of the gas molecules from the pores.

### Crystal structure of the contracted phase

**Figure 3:**
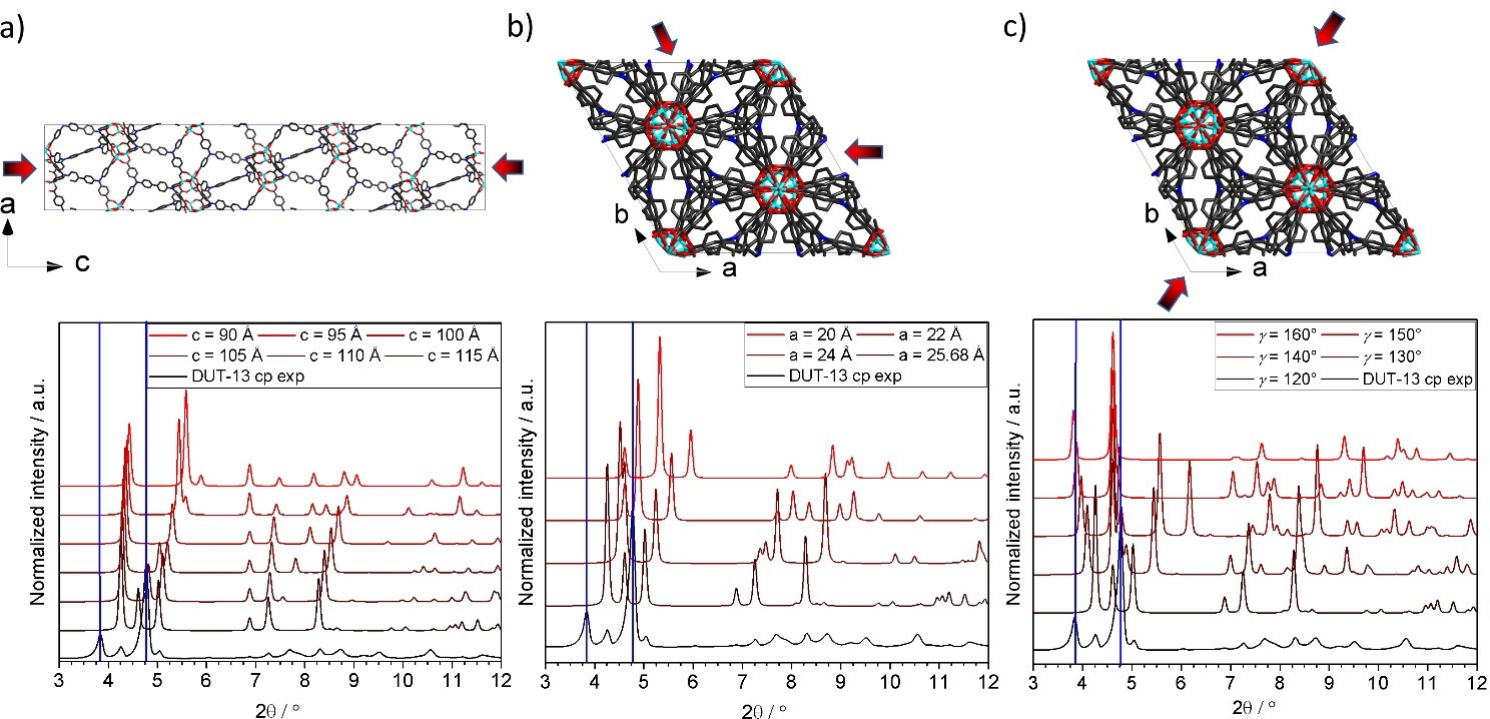
Possible breathing scenarios in DUT‐13 and the corresponding PXRD patterns: a) Contraction along the *c*‐axis. b) Contraction along the *a‐* and *b*‐axes. c) Contraction along the [110] diagonal.

**Figure 4:**
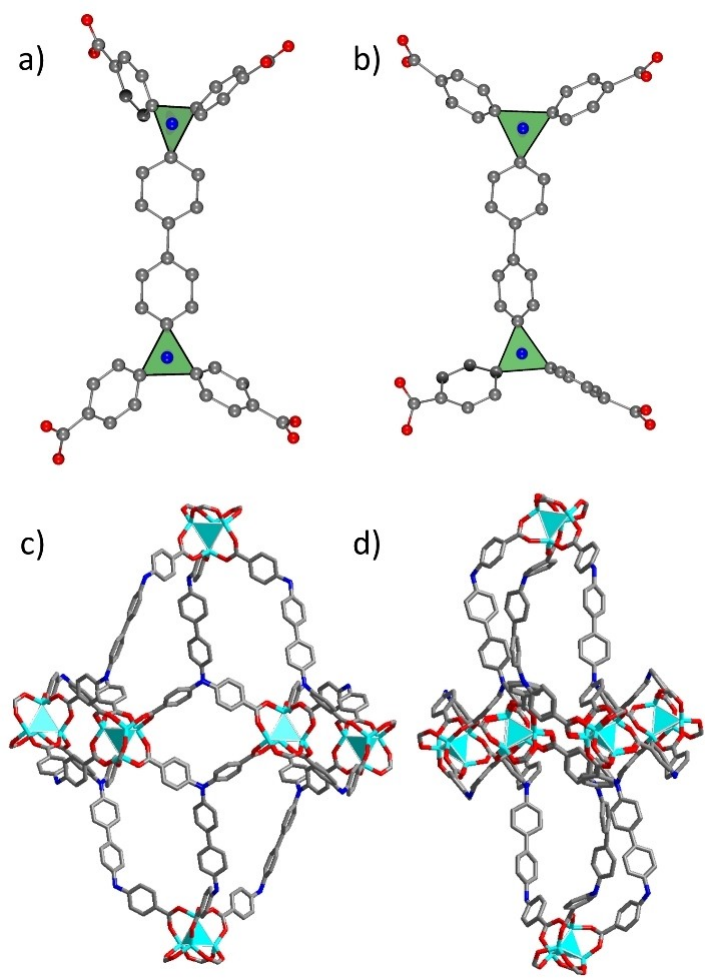
Conformations of the benztb^4−^ linker in the a) *op* and b) *cp* phases and contraction of the octahedral pore upon transition from c) *op* to d) *cp* phase.

Firstly, we aimed to solve the crystal structure of the *cp* phase of DUT‐13. From the first report on DUT‐13 it is known that after nitrogen physisorption the MOF is in a different phase than before the experiment, presumably in the *cp* phase or a mixture of *op* and *cp* phases.^[11a]^ Therefore, the experiment was reproduced and high‐resolution PXRD was measured. However, the ab initio solution of the crystal structure was not possible because of the phase mixture and low number of reflections in PXRD patterns (Figure S2). Due to this reason, an initial structural model for Rietveld refinement could only be derived by manually applying a reverse Monte Carlo (RMC) approach. For this purpose, we analysed several scenarios of the unit cell distortions, which can lead to a structural contraction by 1) a decrease in the length of the *c*‐axis (Figure 3a), 2) a decrease in the length of the *a‐* and *b*‐axes (Figure 3b) and 3) an increase in the *γ*‐angle (Figure 3c). Due to the absence of appropriate code for automatic analysis, the structural models were first optimized using the geometry optimization tool of the *Materials Studio 5.0* software with implemented UFF (universal force field). In the case of scenario (c), the coarse matching of the theoretical and experimental patterns was observed at *γ*=150° and therefore the corresponding structure was chosen as a starting model for the Rietveld refinement. The crystal structure was refined in the monoclinic space group *C2/c*, which is the subgroup of the *R* $\bar{3}$ *c* group of index k3. The asymmetric unit contains 1.5 benztb^4−^ ligand molecules and one Zn_4_O^6+^ cluster. The detailed comparison of these two structures illustrates the contraction mechanism of DUT‐13. Comparison of the N⋅⋅⋅N distances within the ligand molecules indicates slight elongation from 9.93 Å in *op* phase to 10.23–10.39 Å in the *cp* phase (Figure 4).

The C−N−C valence angles and their sum are one of the main measures of distortion in the benztb^4−^ linker and can be used for defining of the out of plane distortions of the sp^2^‐hybridized nitrogen. The sum value of 360° corresponds to the ideal trigonal‐planar configuration, whereas ideal tetrahedral environment results in 328.5°. In the *op* structure the values of C−N−C angles are ranging from 119.2° to 121.3° resulting in a corresponding sum of 359.5° and therefore are close to a trigonal‐planar configuration. The C−N−C angles in the *cp* structure show different values in two symmetrically independent ligand molecules. In the first ligand molecule, the values of C−N−C angles are ranging from 115.8° to 124.4° and the sums of adjacent angles around the two nitrogen atoms are 358.6° and 360.0°, indicating undistorted trigonal geometry of the nitrogen atoms. The second benztb^4−^ linker shows a much higher distortion with 109.5° as the smallest C−N−C angle, which is very close to the tetrahedral value. The sum of all three C−N−C angles amounts to 355.2°, indicating a higher degree of distortion in comparison to the other linker. However, both discussed parameters do not show significant changes, which would lead to a strong structural contraction. As a next step, the ligand conformation was analysed in detail. It is known that the benztb^4−^ ligand assumes various conformations: staggered or eclipsed. In the *op* phase of DUT‐13 the staggered conformation was confirmed by single crystal X‐ray diffraction analysis. The interplanar angle between the two planes defined by the C_3_N moieties amounts to 52.1°. The analysis of the same parameter in the *cp* structure results in 16.4 and 14.0° for two symmetrically independent ligands, indicating an eclipsed conformation of the ligand in the *cp* phase. In summary, it can be concluded that the flexibility mechanism in DUT‐13 is mainly based on the switching between staggered (*op* phase) and eclipsed (*cp* phase) conformations of the ligand, which differs from DUT‐49, where the flexibility is mainly originating from the ligand buckling. Additionally, the distortions at the soft carboxylate‐node hinges and phenyl rings are also contributing to the switching mechanism of the structure of DUT‐13. Despite the structural contraction, DUT‐13*cp* still shows 64.5 % accessible void in the unit cell, which is about 80 % of the initial op phase that has 82.3 %. This difference in the crystallographic porosity motivated us to investigate the geometrical surface area and porosity of these structures.

### Analysis of the porosity of the op and cp phases

The total pore volume is straightforward to derive from the experimental adsorption isotherm and is a valid parameter for comparison between the observed experimental porosity and the porosity of proposed *op* and *cp* structures. Pore volumes were calculated using Zeo++ v.0.3^[18]^ using a probe size of 0.1 Å. Theoretical pore volumes were calculated to be 2.190 and 1.100 cm^3^ g^−1^, for the *op* and *cp* phases, respectively. Experimental values were determined from the isotherms shown in Figure S3. The total pore volume of 2.03 cm^3^ g^−1^ for the *op* phase was determined in the plateau after reopening of the structure at *p*/*p*_0_=0.8 and the total pore volume of 0.91 cm^3^ g^−1^ for the *cp* phase was extracted from the isotherm of the second run at *p/p_0_* =0.2, where the existence of predominantly *cp* phase is expected. In both cases the ratio between experimental and theoretical values remain constant, indicating that ∼90 % of the theoretical value is reached for both phases (Figure S9). Further analysis of the proposed *op* and *cp* structures were undertaken employing grand canonical Monte Carlo simulation using the RASPA2 software.^[19]^ The total nitrogen gas adsorbed at 77 K and 1 atm was simulated using the TraPPE and UFF parameters to describe the interactions.^[20]^ The amount of substance adsorbed was found to be 59.8 and 26.8 mmol g^−1^ for the *op* and *cp* structures, respectively. The simulated quantities fit well with the experimental N_2_ isotherms. We also computed the pore size distribution for both structures. The pore size distributions demonstrate the larger pore of 1.85 nm, present in the *op* phase, collapses and the *cp* phase shows a relatively narrow pore size distribution in the range 0.7–1.0 nm (Figure S9, see also the Supporting Movie).

### Screening for NGA transitions by using methane adsorption at variable temperatures

**Figure 5:**
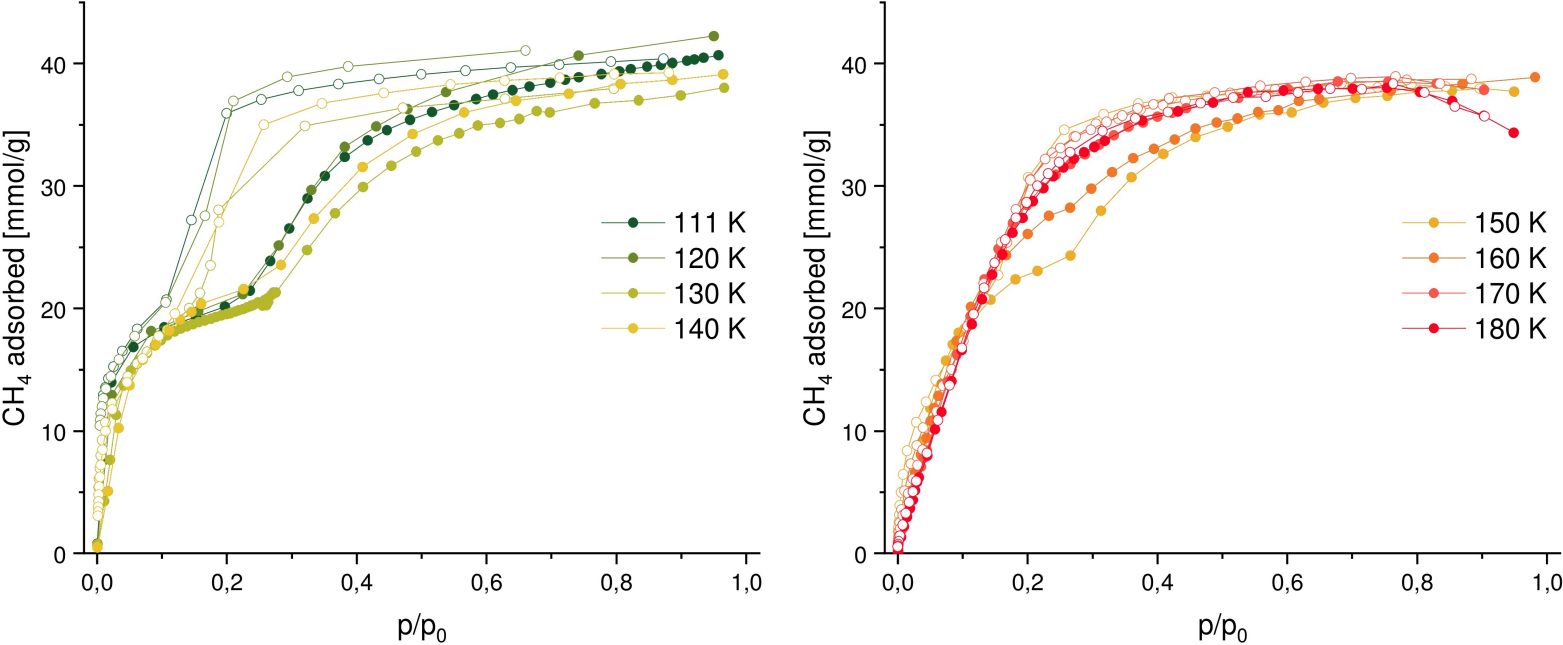
Methane adsorption on DUT‐13 at temperatures from 111 to 180 K.

Recently, we extensively analysed the low and high temperature limits of adsorption‐induced structural contraction in DUT‐49 using methane adsorption as an example and observed an excellent correlation with the computationally predicted upper temperature limit, derived from the osmotic ensemble on a series of GCMC modelled isotherms.^[10]^ As the developed technique allows the stiffness of the framework to be evaluated directly from the adsorption experiments, we conducted a series of methane adsorption experiments on DUT‐13 over the temperature range 111–190 K (Figure 5). Over all temperatures, the methane uptake remains nearly unchanged at around 40 mmol g^−1^. Small changes might be occurring due to the use of a fresh sample for each measurement below 160 K. This approach was used to prevent loss of uptake, as a loss was observed in nitrogen adsorption (Figure S5), in a second physisorption run after a phase transition occurred in the first run. Though the methane uptake is lower than the nitrogen uptake (50 mmol g^−1^), it is interesting to note that the hysteresis in methane adsorption is comparatively wider indicating a better stabilization of the *cp* phase. This is most likely due to the larger amount of methane adsorbed and therefore stronger capillary forces.

At lower temperatures, starting from the boiling point of 111 up to 140 K, a wide hysteresis can be observed, which is a sign of the framework flexibility. Going to higher temperatures the isotherms at 150 and 160 K show a decreasing width of the hysteresis. At 170 and 180 K, reversible isotherms point out a rigid behavior. The temperature dependence of NGA and flexible behavior on DUT‐49 was recently studied and the authors showed a loss of flexibility during methane adsorption at *T*≥135 K.^[10]^ This temperature characteristic supports the assumption that DUT‐13 is a softer network than DUT‐49, with a smaller free energy difference between *op* and *cp* phases. Since a sufficient energy barrier is a requirement for a metastable *op* phase and occuring NGA, this barrier seems to be too low in the case of DUT‐13 to support NGA. Comparing the two networks DUT‐13 and DUT‐49, apart from the resulting topology, two major differences are evident: 1) The metal cluster is a Zn_4_O(CO_2_)_6_ cluster instead of a copper paddlewheel; and 2) the linker does not have a connection of the outer phenyl rings except for the nitrogen. This missing bond allows more rotational freedom around the nitrogen atom in the linker and might be the main reason for a softer network, that cannot show NGA transitions. A stiffening of the linker in that area therefore seems to be crucial for the design of linkers leading to NGA networks.

## Conclusion

Guest‐induced flexibility in the framework DUT‐13 was investigated in situ to analyse the breathing mechanism upon physisorption of nitrogen (77 K) and *n*‐butane (273 K). The crystal structure of the *cp* phase, solved from PXRD data by using a computation‐assisted semiempirical approach, shows a pore volume only half that of the *op* phase; this is consistent with the corresponding isotherms. The contraction mechanism is mainly based on the conformational isomerism of the benztb^4−^ linker, which transforms from a staggered conformation in the *op* phase to a more eclipsed one in the *cp* phase, leading to contraction of the larger pore.

A nearly complete *op*→*cp*→*op* transition was observed in case of *n*‐butane adsorption at 273 K, whereas in the case of weakly interacting nitrogen molecules, a portion of the sample remains in the *op* phase over the entire pressure range. Apparently, in case of DUT‐13, the contraction is crystallite‐size‐dependent, as with a number of other switchable MOFs;^[7c]^ this should be investigated more in detail in the future.

Methane adsorption at various temperatures showed a wide hysteresis between 111 and 140 K. The hysteresis width decreases until it disappears completely at 170 K leading to a reversible isotherm, typical for rigid frameworks. The fact that breathing is observed over a broader temperature range than with DUT‐49^[10]^ demonstrates that thermodynamics and kinetics favor the DUT‐13 contraction. The linker and hinges in DUT‐13 are not stiff enough to support the metastable states required for NGA.

## Experimental Section

**General methods**: All chemicals and solvents used in the syntheses were at least of reagent grade and were used without further purification.

PXRD patterns were collected in transmission geometry with a *STOE STADI P* diffractometer operated at 40 kV and 30 mA with monochromatic Cu${}_{K\alpha {}_{1}}$ (*λ*=0.154059 nm) radiation, a scan speed of 120 s/step and a detector step size of 2*θ*=6°. The samples were placed between no‐diffracting adhesive tape. “As made” samples were analysed while suspended in DMF. Desolvated samples were prepared in a glove box under argon atmosphere. Theoretical PXRD patterns were calculated based on crystal structures using *Mercury 3.9* software package.

SEM images of the DUT‐13 samples were taken with secondary electrons in a *HITACHI SU8020* microscope using 2.0 kV acceleration voltage, a working distance of 10.5 mm and different magnifications. The samples were prepared on a sticky carbon sample holder. To avoid degradation upon exposure to air, the samples were prepared in a glove box under argon atmosphere.

Nitrogen physisorption was measured at 77 K on a Quantachrome Quadrasorb SI automated surface area and pore size analyser.

Methane adsorption isotherms in the broad range of pressures and temperatures were collected using a home‐built system constructed from a volumetric BELSORP‐HP (Microtrac MRB) device and a closed‐cycle helium cryostat DE‐202D (ARS‐Cryo) in the self‐made high‐pressure adsorption cell based on a 1/2 inch VCR parts. The adsorption system is designed for static physisorption experiments in the temperature range of 4 to 300 K and a pressure range of 0.01 to 8000 kPa. The *BELSORP‐HP* instrument is equipped with a turbomolecular pump and possesses a temperature‐controlled standard volume (*V_s_*) of 20.663 cm^3^. The measurement cell was calibrated at each temperature ensuring precise measurements of the adsorbed gas amount. The dead volume of the system was determined using helium gas with 99.999 % purity. Methane of 99.999 % purity was used in all adsorption experiments. All adsorption isotherms were measured using equilibrium conditions of 0.1 % of pressure change within 500 s.

***In situ*****PXRD during the physisorption of nitrogen and*****n*****–butane**: In situ PXRD patterns upon physisorption of nitrogen at 77 K and *n*‐butane at 273 K were measured at KMC‐2 beamline of BESSY II synchrotron, operated by Helmholtz‐Zentrum Berlin für Materialien und Energie.^[21]^ Customized automated instrumentation, based on the volumetric adsorption instrument and closed‐cycle Helium cryostat, equipped with adsorption chamber with beryllium domes was used in all experiments.^[22]^ PXRD patterns were measured at constant wavelength *λ*=1.54059 Å (*E*=8048 eV) in transmission geometry. Diffraction images were collected using 2*θ* scans and Vantec 2000 detector (Bruker). Each 2D image was measured with 31 s exposure. 15 mg of DUT‐13 powder was used in experiment. Reflections from the crystalline Be‐dome were eliminated by tungsten slits with 5 mm aperture, mounted on the detector cone. The obtained diffraction images were integrated by using Datasqueeze 2.2.9^[23]^ with further processing in FITYK 0.9 software.^[24]^ Adsorption equilibrium setting was defined as pressure change of 0.1 % within 300 s.

**Rietveld refinement of the DUT‐13** ***cp*** **phase**: The starting structural model for Rietveld refinement was derived empirically from the DUT‐13 *op* phase by stepwise increasing of the *γ*‐angle in the unit cell with artificially reduced space group symmetry from *R* $\bar{3}$ *c* to *P1*. The search of the additional symmetry in the most suitable model resulted in the space group *C*2/*c*, which reduced the asymmetric unit to one Zn_4_O cluster and 1.5 benztb linker molecules. The Rietveld refinement was performed using Reflex tool, implemented in Materials Studio 5.0 software.^[25]^ The reflection at *2θ*=4.3° in PXRD patterns belongs to the DUT‐13 *op* phase and was omitted from the refinement. A treatment of all phenyl rings and carboxylates as rigid bodies allowed the number of motion groups to be significantly reduces, and final refinement was performed by using Rietveld with energy option (1 % UFF contribution) using 23 motion groups, which resulted in 114 degrees of freedom. The final Rietveld refinement plot is given in Figure S8 and the main experimental parameters in Table S1.

**General procedure for the synthesis of DUT‐13**: 446.2 mg (2.03 mmol, 13.5 equiv) zinc acetate dihydrate were dissolved in 12.5 mL *N*,*N*‐dimethylformamide (DMF) while 100.2 mg (0.15 mmol, 1.0 equiv) H_4_benztb were dissolved in 7.5 mL *N*‐methyl‐2‐pyrollidone (NMP). The zinc solution was distributed among two Pyrex tubes before the linker solution was added. After closing the tubes, the reaction was carried out in a drying oven at 80 °C for one week. The yellow crystals were washed with DMF for several times and then dried and activated using supercritical carbon dioxide in a Jumbo Critical Point Dryer 13200 J AB after solvent exchange from DMF to acetone.

## Conflict of interest

The authors declare no conflict of interest.

## Supporting information

As a service to our authors and readers, this journal provides supporting information supplied by the authors. Such materials are peer reviewed and may be re‐organized for online delivery, but are not copy‐edited or typeset. Technical support issues arising from supporting information (other than missing files) should be addressed to the authors.

Supplementary

Supplementary

Supplementary

Supplementary

Supplementary
