# Supplementary figures and images for: Unraveling the Guest‐Induced Switchability in the Metal‐Organic Framework DUT‐13(Zn)
Source: Chemistry. 2021 May 21;27(37):9708–15. doi: 10.1002/chem.202100599 (PMC8362161; doi:10.1002/chem.202100599)

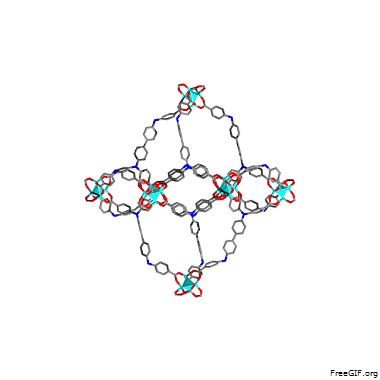

Supplement: Supplementary file 1 — Supplementary [file CHEM-27-9708-s002.gif]

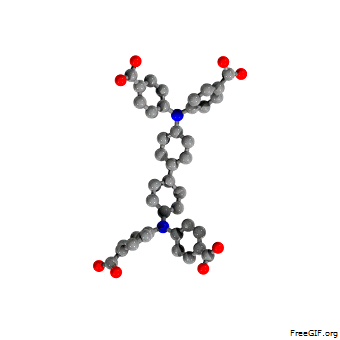

Supplement: Supplementary file 2 — Supplementary [file CHEM-27-9708-s005.gif]

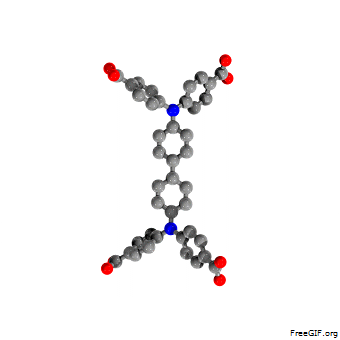

Supplement: Supplementary file 3 — Supplementary [file CHEM-27-9708-s003.gif]

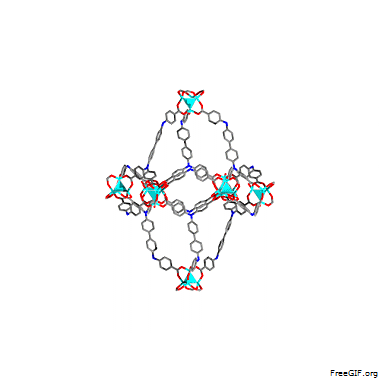

Supplement: Supplementary file 4 — Supplementary [file CHEM-27-9708-s001.gif]
