# Supplementary material for: Unraveling the Guest‐Induced Switchability in the Metal‐Organic Framework DUT‐13(Zn)
Source: Chemistry. 2021 May 21;27(37):9708–15. doi: 10.1002/chem.202100599 (PMC8362161; doi:10.1002/chem.202100599)
Supplement: Supplementary file 5 — Supplementary [file CHEM-27-9708-s004.pdf]

Chemistry–A European Journal

Supporting Information

Unraveling the Guest-Induced Switchability in the Metal-Organic Framework DUT-13(Zn)

Bodo Felsner, Volodymyr Bon,* Jack D. Evans, Friedrich Schwotzer, Ronny Grünker, Irena Senkovska, and Stefan Kaskel*

1. PXRD patterns 2

2. Nitrogen physisorption 3

3. SEM analysis 5

4. Rietveld analysis of DUT-13*cp* 6

5. Pore size distribution analysis 7

1. PXRD patterns

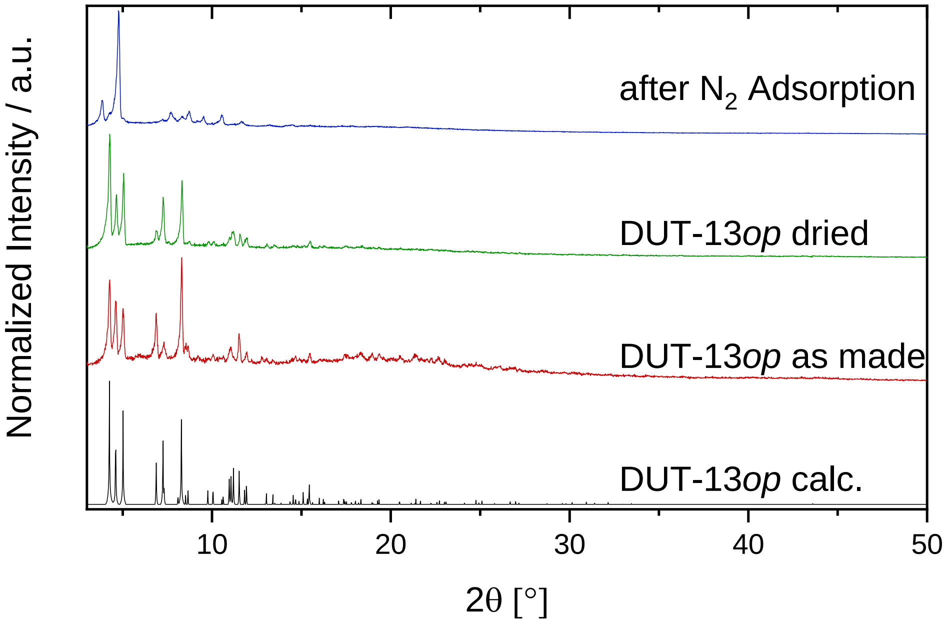

Figure S1: PXRDs of DUT-13: Theoretical pattern calculated from the single crystal XRD structure, as made phase in DMF, supercritically dried and after one run of nitrogen adsorption. 2Theta range from 3 to 50°.

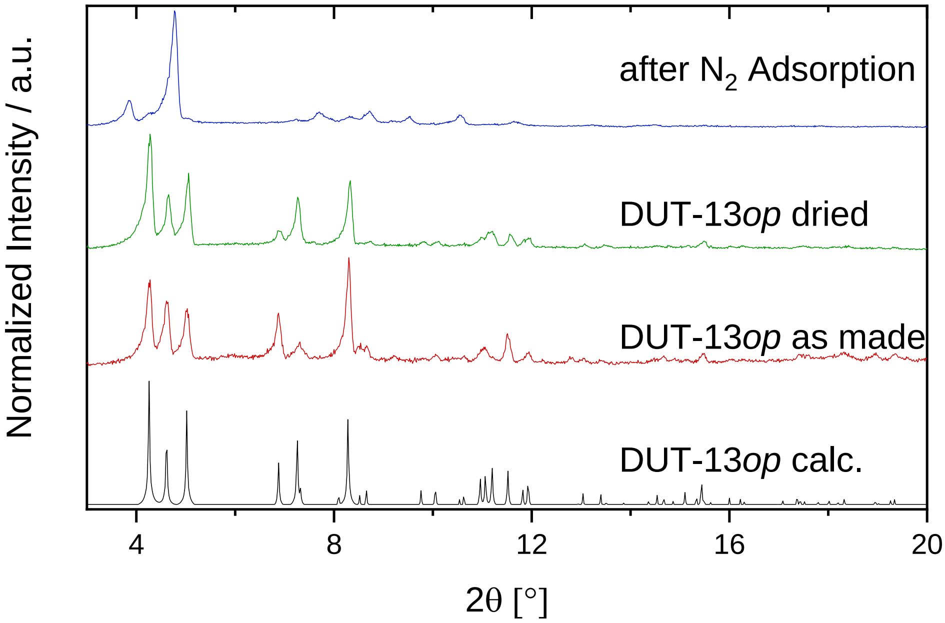

Figure S2: PXRDs of DUT-13: Theoretical pattern calculated from the single crystal XRD structure, as made phase in DMF, super critically dried and after one run of nitrogen adsorption. 2Theta range from 3 to 20°.

2. Nitrogen physisorption

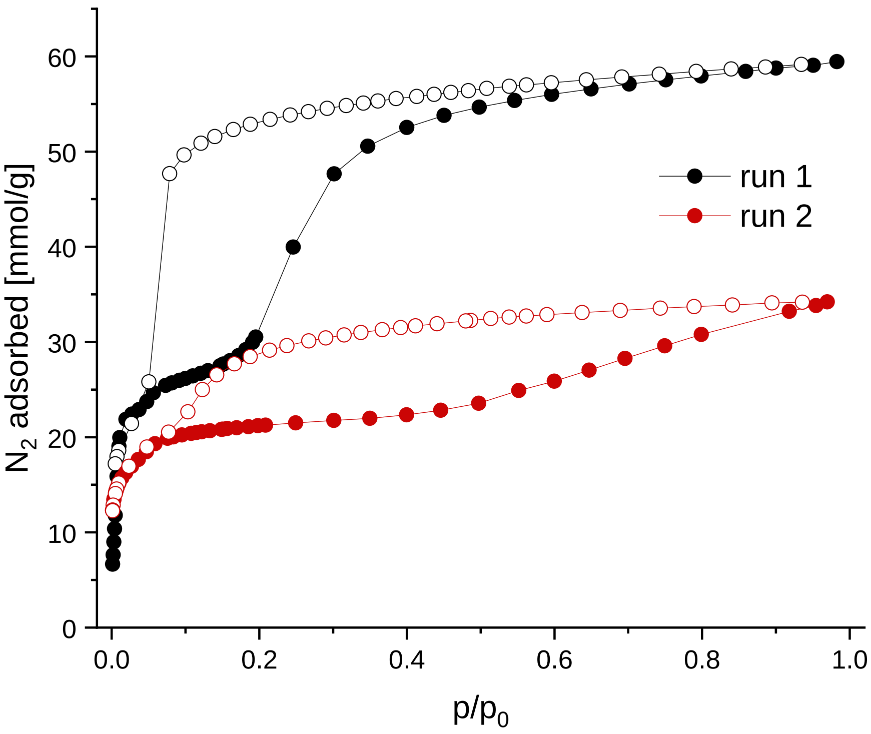

Figure S3: N_2 physisorption of DUT-13. Run 1 after supercritical drying and activation under vacuum at 100 °C. Run 2 without further activation after measurement of run 1.

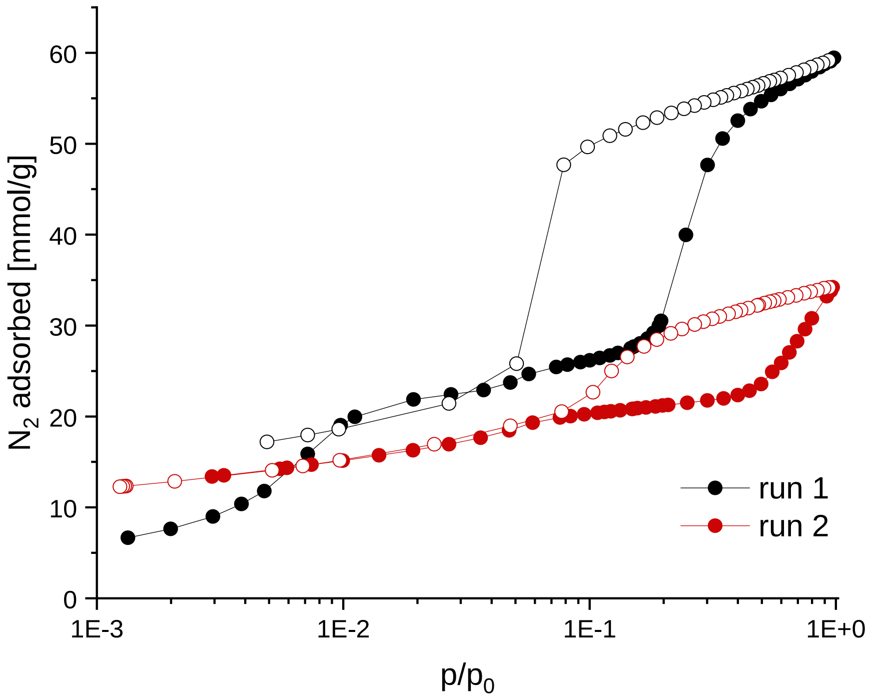

Figure S4: N_2 physisorption of DUT-13 in semilogarithmic plot. Run 1 after supercritical drying and activation under vacuum at 100 °C. Run 2 without further activation after measurement of run 1.

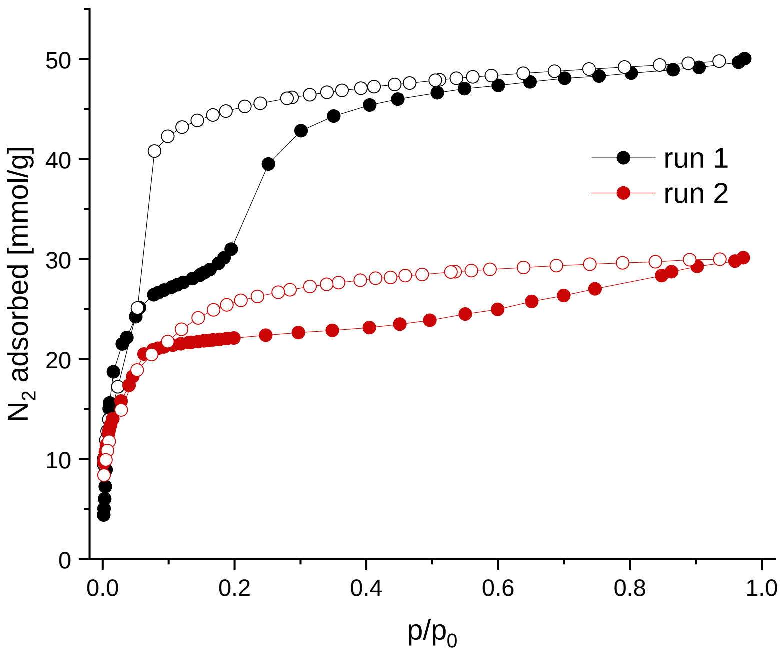

Figure S5: N_2 physisorption of the DUT-13 batch used for CH_4 adsorption measurements. Run 1 after supercritical drying and activation under vacuum at 100 °C. Run 2 without further activation after measurement of run 1.

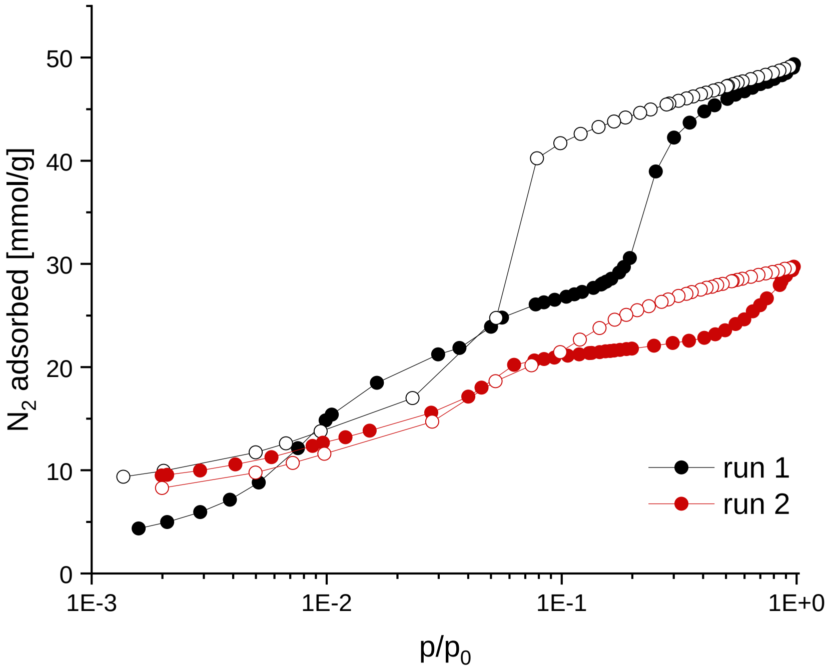

Figure S6: N_2 physisorption of the DUT-13 batch used for CH_4 adsorption measurements in semilogarithmic plot. Run 1 after supercritical drying and activation under vacuum at 100 °C. Run 2 without further activation after measurement of run 1.

3. SEM analysis

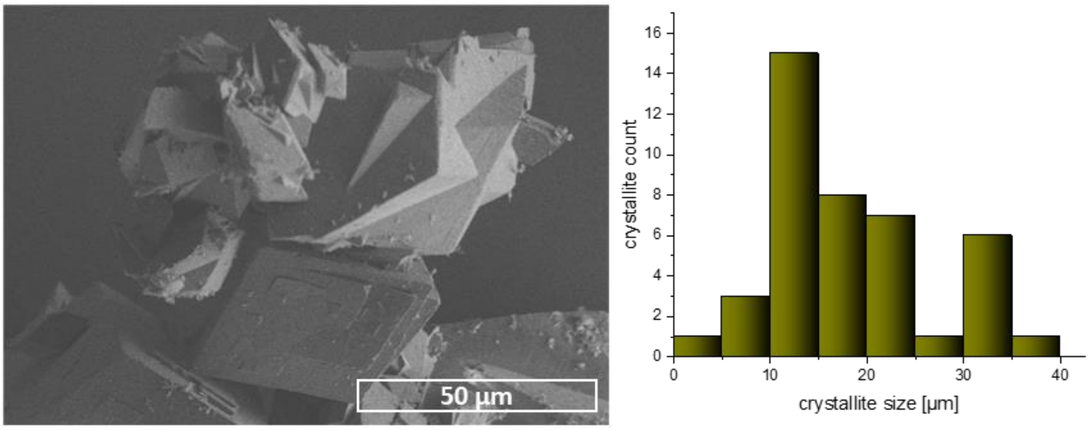

Figure S7: SEM image of the DUT-13 batch and its crystal size distribution.

4. Rietveld Refinement of DUT-13*cp*

Table S1: Rietveld Refinement of the DUT-13*cp* structure.

	**DUT-13*cp***
Formula unit	$C_{60}H_{36}N_3O_{13}Zn_4$
Z	8
Symmetry, space group	Monoclinic, C2/c
a (Å)	50.1986(312)
b (Å)	13.84186(645)
c (Å)	40.45758(133)
β °	113.361(75)
Unit cell volume, (Å^3)	25807.2
Wave length (Å)	1.54059
2θ range (°)	3 - 30
Profile function	Thompson-Cox-Hastings
U	0.63222
V	0.01203
W	0.00076
X	0.20427
Y	0.09309
Asymmetry correction	Berar-Baldinozzi
P1	-0.04822
P2	-0.00991
P3	-0.00027
P4	0.00006
Final R_{wp}	0.2044
Final R_p	0.1415

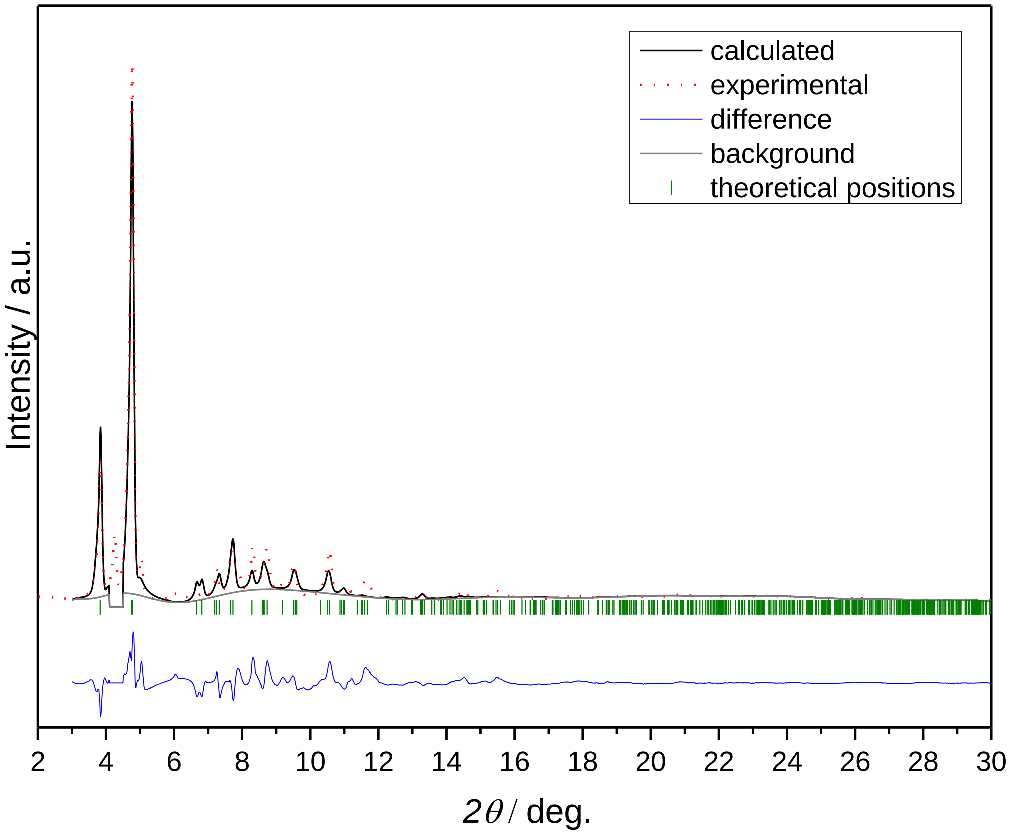

Figure S8: Rietveld plot for DUT-13*cp*.

5. Pore size distribution analysis

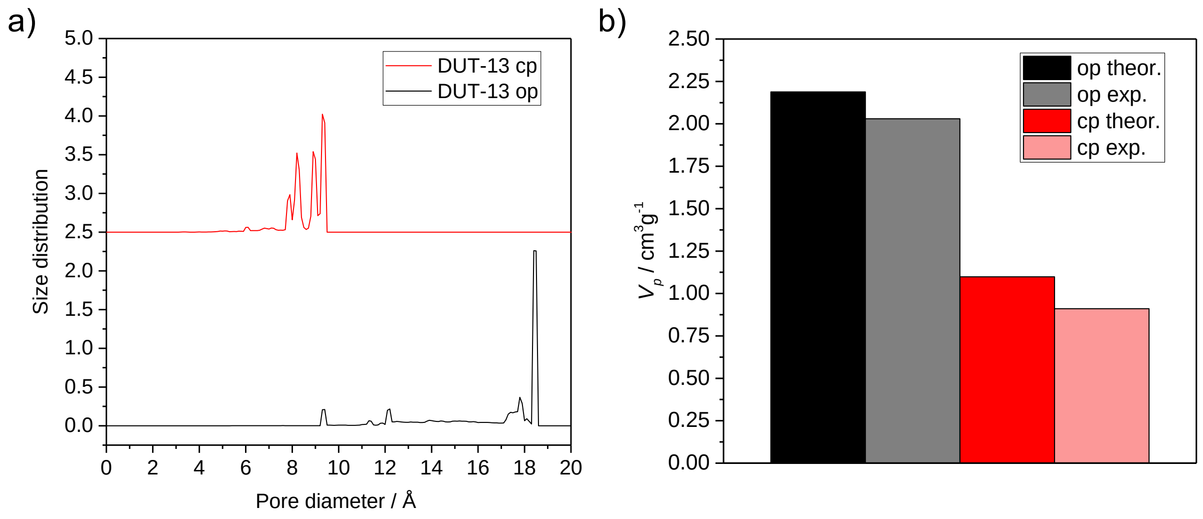

Figure S9: a) Pore size distribution and b) pore volume for DUT-13*op* and *cp* phases.
